# Antigenotoxicity and antimutagenicity of ethanolic extracts of Brazilian green propolis and its main botanical source determined by the *Allium cepa* test system

**DOI:** 10.1590/1678-4685-GMB-2015-0130

**Published:** 2016-05-24

**Authors:** Matheus Mantuanelli Roberto, Cláudia Masrouah Jamal, Osmar Malaspina, Maria Aparecida Marin-Morales

**Affiliations:** 1 Departamento de Biologia, Instituto de Biociências, Universidade Estadual Paulista "Júlio de Mesquita Filho", Rio Claro, SP, Brazil; 2 Departamento de Ciências Farmacêuticas, Centro de Ciências da Saúde, Universidade Federal do Espírito Santo, Vitória, ES, Brazil; 3 Centro de Estudo de Insetos Sociais, Universidade Estadual Paulista "Júlio de Mesquita Filho", Rio Claro, SP, Brazil

**Keywords:** chromosomal aberration, micronucleus, anticlastogenicity, protective effects, flavonoids

## Abstract

Brazilian green propolis is a resinous substance prepared by bees from parts of the plant *Baccharis dracunculifolia*. As it possess several biological properties, this work assessed the cytotoxic/anticytotoxic, genotoxic/antigenotoxic and mutagenic/antimutagenic potential of ethanolic extracts of Brazilian green propolis (EEGP) and of *B. dracunculifolia* (EEBD), by means of the *Allium cepa* test system. The effects were evaluated by assessing the chromosomal aberrations (CA) and micronuclei (MN) frequencies on meristematic and F1 generation cells from onion roots. Chemical analyses performed with the extracts showed differences in flavonoid quality and quantity. No genotoxic or mutagenic potential was detected, and both extracts were capable of inhibiting cellular damage caused by methyl methanesulfonate (MMS) treatment, reducing the frequencies of CA and MN. By these data, we can infer that, independent of their flavonoid content, the extracts presented a protective effect in *A. cepa* cells against the clastogenicity of MMS.

## Introduction

Over the last 30 years, the pharmacological and chemical properties of propolis became the aim of intensive studies, and since the end of the 20th century, the paradigm related to the chemistry of propolis has changed drastically. By the 1960s, it was known that propolis is chemically complex, but it was considered as chemically stable, like beeswax and bee venom (Bankova, 2005a). In recent years, however, the analysis of several propolis samples from different regions of the world showed that the chemical composition is highly variable (Bankova, 2005a). Recently, propolis, especially from Brazil, has attracted both commercial and scientific interest (Kumazawa *et al.*, 2003).

Propolis is a resinous substance collected and prepared by bees from several parts of plants. During preparation, the bees add saliva enzymes to a partly digested material, and they mix this with wax and use it in the building of their hive (Bankova *et al.*, 2000). In order to produce propolis, the bees use material from several parts of plants and in different stages of development. Plants, for example, actively secrete substances found in propolis in the form of exudates from a wound. They also secrete lipophilic materials of leaves, and also buds, resins, latex and others. Hence, the complexity and the chemical variety of propolis are deeply related to the ecoflora of the region which the bees commonly visit (Burdock, 1998). Not surprisingly, around 300 substances have been identified in different samples of propolis, the main ones being phenolic compounds. The majority of these substances belongs to three major groups: flavonoids, phenolic acids, and esters, with concentrations varying according to the local ecoflora (Simões *et al.*, 2004).

Propolis is not only a building material, but also an important chemical defense weapon used by bees against pathogenic microorganisms. Humans have been using propolis as a remedy since ancient times, and nowadays it still is most frequently consumed to treat wounds, burns and stomach ulcers, also using it as a mouth rinse (Bankova, 2005b). According to Banskota *et al.* (2001), propolis can be used in popular medicine and in the preparation of food and beverages.

The constituents of propolis and their activity can differ substantially according to the method of extraction used, and many authors suggested several biological properties, from anti-inflammatory to anticarcinogenic (Marcucci, 1995; Burdock, 1998; Castaldo and Capasso, 2002; Menezes, 2005; Salatino *et al.*, 2005; Santos Pereira *et al.*, 2002).

As the genomes of all living organisms are subjected to damage by external agents and endogenous processes, genotoxic effects may occur that hamper DNA integrity and compromise the function of genes. If some genes, or a whole chromosome, suffer permanent damages, a mutation may become established, resulting in a heritable modification of certain characteristics. Antimutagenic agents are known to counteract the effects of mutagenic agents, and there is a high demand to identify such compounds (Sloczynska *et al.*, 2014). According to some authors (De Flora and Ferguson, 2005; Steward and Brown, 2013; Tatsuzaki *et al.*, 2014), the daily consumption of antimutagenic agents could prevent cancer and genetic diseases in humans. For example, Varanda *et al.* (1999) have shown an antimutagenic effect exerted by an ethanolic extract of propolis on *Salmonella typhimurium* (TA102, TA100 and TA98). The effect was observed against the mutagens daunomycin (TA102), benzo[a]pyrene (TA100) and aflatoxin B1 (TA98). The authors concluded that the antimutagenic effect is due to the presence of flavonoids, compounds of recognized antioxidant activity (Varanda *et al.*, 1999).

Propolis from temperate regions contains mainly phenolic compounds, including flavonoids and cinnamic acid derivatives, while diterpenes and prenylated compounds are absent (Bankova *et al.*, 2000). On the other hand, such latter compounds are present in samples of propolis from tropical regions, mixed with lignans, flavonoids and other classes of phenolic compounds. The difference between these regions, and consequently between the two types of propolis, are due to their divergent flora (Bankova *et al.*, 2000).

Since propolis is a complex mixture of substances, and because Brazilian green propolis has specific compounds, there has been an effort to know the chemical profiles, to assess which plant is the main botanical source of propolis from tropical regions. Different studies using different methods have identified *Baccharis dracunculifolia* as the source for bees to produce the Brazilian green propolis type (Kumazawa *et al.*, 2003; Park *et al.*, 2004; Teixeira *et al.*, 2005; Verdi *et al.*, 2005). This type of propolis is endemic, original from the south of the state of Minas Gerais and from the north of the state of São Paulo, in Brazil, where it is possible to find *B. dracunculifolia*, also known as "*alecrim-do-campo*" or "*vassourinha*" (Park *et al.*, 2004).

The *Allium cepa* test system is recommended for toxicological evaluation, and it has been validated by the World Health Organization, the United Nations Environmental Program, and the United States Environmental Protection Agency (Mauro *et al.*, 2014). According to Leme and Marin-Morales (2009), this test system allows the simultaneous assessment of cytotoxic, genotoxic and mutagenic effects of a determined compound, environmental samples or natural products, without the need of performing different assays. In contrast, other test systems require the analysis of these endpoints separately, by different techniques and exposures. Furthermore, while mammalian (*in vivo* test) and cell culture experiments (*in vitro* tests) are also suggested for evaluating antimutagenicity, the *A. cepa* assay can show the primary effects of natural products at low cost, as it has high sensitivity and shown good correlation with other test systems, such as the Ames test (Rank and Nielsen, 1994), a mammalian test system (Chauhan *et al.*, 1999), a human lymphocyte test system (Fiskesjö, 1985), and carcinogenicity tests in rodents (Rank and Nielsen, 1994), with the advantage of excluding the need for animal use and sacrifice (Leme and Marin-Morales, 2009).

With this in mind, this work aimed to: (1) characterize the chemical composition of propolis and *B. dracunculifolia* extracts in a simple manner; (2) assess the cytotoxic, genotoxic and mutagenic potential of an ethanolic extract of Brazilian green propolis (EEGP) and of an ethanolic extract of its main plant source, *B. dracunculifolia* (EEBD); (3) evaluate the anticytotoxic, antigenotoxic and antimutagenic potential in samples of these extracts. To achieve this, the frequencies of chromosomal aberrations (CA) in meristematic cells, and the frequencies of micronuclei (MN) in meristematic and F1 cells from roots of *Allium cepa* (onion) were analyzed. Finally, we also emphasize that this test system could be applied to investigate the protective effects of natural substances on cells.

## Materials and Methods

### Propolis: origin and its botanical source

The samples of Brazilian green propolis and of *B. dracunculifolia* were provided by the apiary Apiário Floresta Comércio Importação e Exportação Ltda., which were collected in the surroundings of the municipality of Carvalhópolis (21°46′42″S / 45°50′30″W), in the south of Minas Gerais, Brazil, in the summer of 2007 (season known as warm and wet). The samples were collected and conditioned in a closed recipient and protected from light until processing.

### Preparation of ethanolic extracts

Ethanolic extracts were prepared at the Laboratório de Produtos Apícolas do Centro de Estudos de Insetos Sociais of the Universidade Estadual Paulista (UNESP), in Rio Claro, Brazil. The extraction was made according to the standard procedure for propolis, with ethanol as the solvent. Ethanol is the most frequently used solvent, because it possesses a great extraction capacity, removing around 50-70% of the propolis components, while the aqueous extraction method removes only around 10% (Sforcin and Bankova, 2011).

The ethanolic extract of Brazilian green propolis (EEGP) was prepared using the following method: 30 g of crude propolis were crushed, and then 70% ethanol was added until the mixture reached 100 mL of volume. This established a proportion of 30% of propolis and 70% of ethanol. This mixture was put into a flat-bottomed flask and then submitted to a process known as hot extraction under simple reflux for 6 h. After this period, the extract was filtered while still hot, chilled and put into a 100 mL graduated cylinder, protected by foil, to decant the suspended wax. After the wax precipitation, the extract was filtered again to remove the wax and then, finally, the volume was completed with 70% ethanol to correct the ethanol loss due to evaporation.

The ethanolic extract of Baccharis dracunculifolia (EEBD) was prepared similar to the preparation of EEGP, but instead of propolis, 30 g of young leaves and buds of B. dracunculifolia were used, which were dried and macerated. Subsequently, the same procedure was used, including the decantation part, to simulate the same loss of ethanol.

### Phytochemical characterization of the ethanolic extracts

The presence of phytochemical compounds like alkaloids, steroids, triterpenes, saponins, coumarins and flavonoids was qualitatively evaluated. The extracts were subjected to pharmacognostic classical tests to detect the presence of metabolite classes (Wagner *et al.*, 1984; Costa, 1986). Prior to the chemical analyses, the ethanolic extracts were dried using a rotary evaporator, and EtOH and CHCl_3_ solutions of the extracts were prepared for the tests.

For the analysis of alkaloids, 1 mL of EtOH solution of the extracts was mixed with 1 mL of HCl (p.a.) and treated with a few drops of Dragendorff's reagent. In this test, the orange precipitation indicates the presence of alkaloids.

To analyze the presence of steroids and triterpenes, 1 mL of chloroform solution of the extracts was mixed with 2 mL of acetic anhydride and treated with three drops of H_2_SO_4_ (p.a.). The change in colour from blue or green indicates the presence of steroids, and the change from reddish-brown indicates the presence of triterpenes.

The presence of saponins was evaluated by the frothing test: 20 mL of EtOH solution of the extracts was mixed with 15 mL of distilled water and 1 mL of saturated solution of sodium carbonated and, then, this mixture was boiled. After boiling, the mixture was filtered and 2 mL was separated using a graduated cylinder. Then, 98 mL of distilled water was added and the solution was shaken vigorously. The formation of stable and persistent froth indicates the presence of saponins.

The presence of coumarins was verified by adding 1 drop of each EtOH solution of extract in a filter paper. After dried, the blots were observed in a UV chamber. In sequence, KOH 10% (w/v) was added upon the blots and they were observed in the UV chamber again. The fluorescence indicates the presence of coumarins.

The presence of flavonoids was assessed by the cyanidin reaction: 1 mL of EtOH solution of the extracts was mixed with 1 mL of HCl (p.a.) and, then, Mg powder was added. A change in colour varying from brown until red indicates presence of flavonoids.

The quantification of total flavonoids was performed based on the quercetin standard, according to Woisky and Salatino (1998), with slight modifications, as follows: 5.0 mL of absolute ethanol were put in a test-tube with 5 μL of each sample, separately, with subsequent addition of 100 μL of AlCl_3_ (2% methanol solution). After 5 s of agitation and 30 min of rest protected from the light, spectrophotometry readings were done at λ = 425 nm.

In order to obtain the chromatographic profiles by thin layer chromatography and to determine the types of simple phenolic compounds and flavonoids present in both extracts, specific revealers and solvents were used for each class of compounds (Wagner and Bladt, 2001).

The total soluble solids (TSS) content present in the extracts was measured by an Abbe's refractometer (Biobrix^®^ 107, Brazil), with the result given in Brix degrees (°Bx). To determine TSS, two drops of crude EEGP or EEBD were placed on the prism of the refractometer, and the intersection of light and dark fields marked the Brix value.

### The *Allium cepa* test system

The biological material used in this study, as a plant test system, to assess the effects of the ethanolic extracts, was based on seeds of *Allium cepa* (2n = 16 chromosomes), obtained from the TopSeed^®^ (Agristar do Brasil Ltda) trademark, from the same batch and variety (Baia Periforme onions). This plant species is indicated for evaluation in genotoxicological studies (Leme and Marin-Morales, 2009).

**Figure 1:**
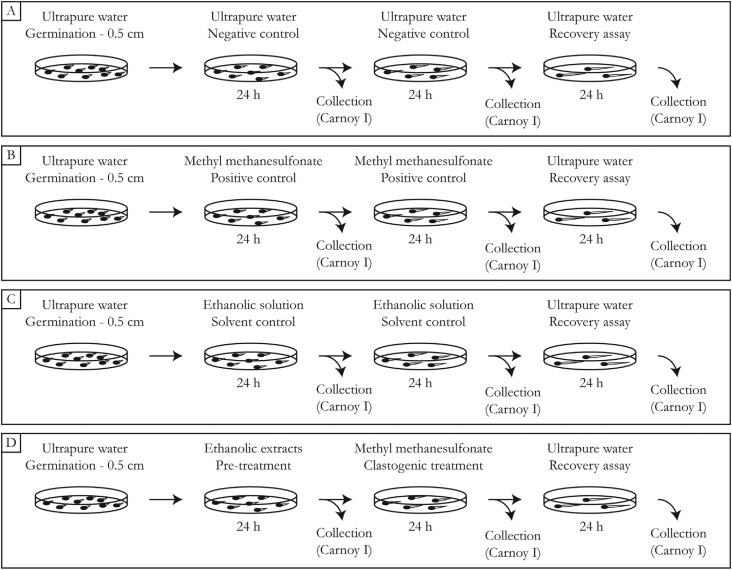
Experimental design. A. Negative control (NC); B. positive control (PC); C. solvent control (SC); D. Pre-treatments with ethanolic extracts, followed by a clastogenic treatment and a recovery assay.

The exposures were performed as shown in Figure 1. Initially, 14 Petri dishes containing 100 seeds each were maintained in an incubator at 22 ± 2 °C, until they reached about 0.5 cm. After this stage, four groups were treated differently, using two plates per treatment and per concentration.

Group A: negative control (NC). The seeds and the roots remained exposed to ultrapure water during the whole experiment. Samples were collected every 24 hours for a period of 72 hours of exposure (total of 3 samples).

Group B: positive control (PC). The germinated roots were exposed to a methyl methanesulfonate (MMS - CAS no. 66-27-3, Sigma-Aldrich) solution at a concentration of 4 × 10^−4^ M. Three collections were made for this group, concomitantly with the NC collections.

Group C: solvent control (SC). The roots were exposed to two ethanolic solutions with concentrations similar to those found in the extracts (SC-1 = 0.042 μL/mL and SC-2 = 0.21 μL/mL, equivalent to the ethanolic extracts concentration of 0.06 μL/mL and 0.30 μL/mL, respectively). The collection followed the same pattern of the NC and PC groups.

Group D: treatments with the ethanolic extracts. In this group, the roots were individually exposed to the extracts for a period of 24 hours. After this time, a part of the roots was collected, while the remainder was transferred to another Petri dish containing a solution of MMS, at the same concentration as the PC. After another period of 24 hours, a part of the roots was collected and fixed, while the other part was transferred to another Petri dish for a recovery assay in ultrapure water for 24 hours. After the recovery period, the material was also collected and fixed.

Thus, for each treatment assay series, we also performed a collection of the control groups (NC, PC and SC). Two independent experiments were performed, in which the exposures were conducted simultaneously, in duplicate, for each experiment. The biological material collected was fixed by a Carnoy I solution (3 parts of ethanol and 1 part of acetic acid - v/v) and stored in a refrigerator at 4 °C until the processing.

For slide preparation, the procedure described by Bianchi *et al.* (2011) was followed, in which the previously fixed root tips were washed in distilled water and hydrolyzed in HCl 1N at 60°C for 8 min. The roots were washed in distilled water again and submitted to a Schiff's reaction for 2 h. Next, the meristematic and F1 regions were cut, covered with a coverslip and carefully squashed into a drop of 2% acetic carmine solution.

Ten slides were prepared per treatment, five from each duplicate, in order to evaluate the presence of chromosomal aberrations and micronuclei, taking into account the percentage of occurrence. About 500 cells from each slide were analyzed, totalling around 5,000 cells per treatment. This same procedure was followed for the F1 regions of the respective meristems. The slides were analyzed by light microscopy (Carl Zeiss Standard Binocular Microscope) at 400 x magnification.

**Figure 2:**
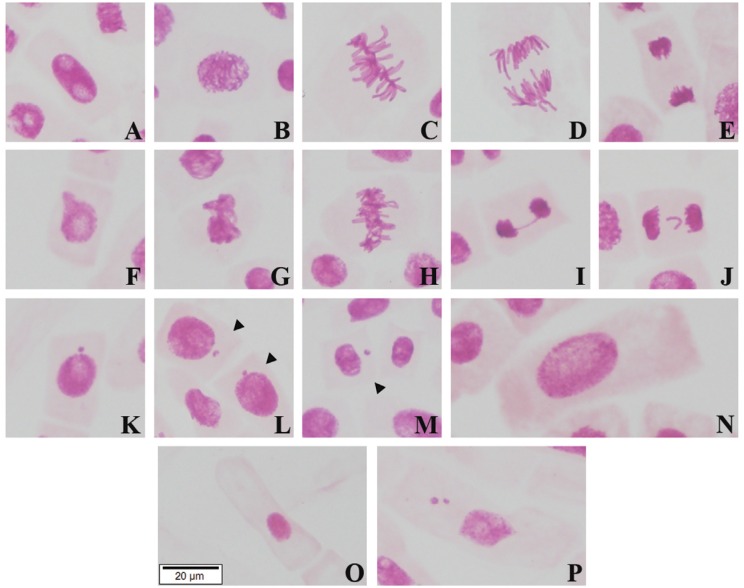
Alterations observed by the *A. cepa* test system analysis. As the treatments with EEGP and EEBD did not induce statistically significantly chromosomal aberrations and micronuclei, these pictures were obtained by the positive control treatment (PC-MMS). A. normal interphase; B. normal prophase; C. normal metaphase; D. normal anaphase; E. normal telophase; F. interphase with a nuclear bud; G. metaphasis with chromosomal adherence; H. polyploid metaphase; I. telophase with a chromosomal bridge; J. telophase with a chromosomal loss; K-M. interphase with micronucleus; N. polyploid interphase; O. normal F1 generation cell; P. F1 generation cell with micronuclei.

Cytotoxic and anticytotoxic effects were evaluated by the mitotic index (MI) calculation, as follows: MI=(total number of cells on division/total number of observed cells)x100 (Leme and Marin-Morales, 2009). Genotoxic and antigenotoxic effects were assessed by the observation and counting of the several types of chromosomal aberrations (CA) seen in meristematic cells, like nuclear buds, binucleated cells, polyploidy cells, chromosomal adherence, C-metaphases, chromosomal bridges, chromosomal loss and breakage, and multipolar anaphases (Leme and Marin-Morales, 2009). Mutagenic and antimutagenic potentials were evaluated by the observation and counting of micronuclei (MN) present on meristematic and on F1 cells (Leme and Marin-Morales, 2009). Antigenotoxic and antimutagenic activities were assessed by the analysis of the percentage of damage reduction for each treatment with EEGP and EEBD, respectively, by the following formula: Reduction (%) = [(*a* - *b*)/(*a* - *c*)]x100 (where: *a* = number of damaged cells in the PC; *b* = number of damaged cells in each treatment; *c* = number of damaged cell in the SC). Examples of alterations in the *A. cepa* test can be observed in Figure 2.

The results obtained were submitted to a D’Agostino & Pearson statistical normality test. As the results did not pass the normality test, we used the non-parametric test of Kruskal-Wallis, followed by the Dunn's multiple comparison tests, with the significance level of p ≤ 0.05.

In order to facilitate the understanding of the results, they will be presented according to the type of substance used in the treatments:

Pre-treatment: based on the first collection, this was important to verify the possible cytotoxicity, genotoxicity and mutagenicity induced by the ethanolic extracts;

Clastogenic treatment: after the pre-treatment, this was performed to evaluate if the ethanolic extracts could protect the *A. cepa* cells against the damages induced by MMS. This was important to assess the anticytotoxicity, antigenotoxicity and antimutagenicity;

Recovery assay: after the clastogenic treatment, the remaining roots were kept in ultrapure water for 24 hours. This is recommended to assess the possible residual effects of the ethanolic extracts on cellular protection, *i.e.*, to evaluate if the extracts could exert their effects only when the cells are being exposed or if the extracts could have an extended activity.

## Results and Discussion

This work assessed the cytotoxic, genotoxic and mutagenic potential of the ethanolic extracts of Brazilian green propolis (EEGP) and of the source plant *Baccharis dracunculifolia* (EEBD), the latter one used in two different concentrations. The anticytotoxic, antigenotoxic and antimutagenic potential of both extracts were also evaluated using the *Allium cepa* test system. The *A. cepa* species (onion) is a well known bioindicator used in environmental monitoring assays (Leme and Marin-Morales, 2009), making this a test system recognized as effective to test environmental samples (Hoshina and Marin-Morales, 2009), as well as general substances and residues (Souza *et al.*, 2009). Several studies using *A. cepa* to evaluate antigenotoxicity and antimutagenicity were published in the last years (Nieva Moreno *et al.*, 2005; Oyeyemi and Bakare, 2013; Felicidade *et al.*, 2014; Mauro *et al.*, 2014), and the present one reinforces that this test system is also efficient to verify the protective effect of substances and natural compounds.

### Phytochemical characterization of the ethanolic extracts

After the phytochemical evaluation, chemical compounds such as cumarins, alkaloids, saponins, steroids and triterpenoids were not found in neither of the ethanolic extracts. It is important to emphasize that it is possible that seasonal changes may occur in the chemical profile of Brazilian green propolis and of *B. dracunculifolia*, so these results are related to the samples collected in the summer of 2007, a period in which the plants had more aerial development and, consequently, were a major resource available for the bees collect the material (resins, young leaves and buds) used to produce propolis.

### Determination of total flavonoids

The total amount of flavonoids present in both extracts was measured by using quercetin as a standard for flavonoids (Woisky and Salatino, 1998). The crude ethanolic extract of Brazilian green propolis (EEGP) showed a concentration of 3.65% (m/m) of flavonoids in its composition, while the crude ethanolic extract of *Baccharis dracunculifolia* (EEBD) showed 0.67% (m/m) of flavonoids, which means about 5.4 less flavonoids than in propolis. From these results, it is possible to see that the highest concentration of EEBD (EEBD-2 = 0.30 μL/mL) used in the assays reaches almost the same amount of flavonoids as the one in the only concentration of EEGP (0.06 μL/mL) tested, allowing to compare the quality of composition.

As honeybees collect several parts of the plant of *B. dracunculifolia* to produce the Brazilian green propolis (Kumazawa *et al.*, 2003; Park *et al.*, 2004), this leads us to infer that bees can concentrate a considerable amount of flavonoids and other phenolic compounds responsible for the wide variety of biological properties exerted by this type of propolis.

The content of flavonoids present in the EEGP is considered high by Brazilian regulations (> 2.0% - m/m) (Brasil, 2001), and this is the main feature that attracts the attention of consumers of several countries worldwide.

### Thin-layer chromatography

Knowing the amount of flavonoids of both extracts, the thin-layer chromatography (TLC) analysis was important to determine the presence of the most frequent classes of secondary metabolites, such as simple phenolic compounds and flavonoids.

The analysis made by TLC, based on comparing the retention factor (RF) obtained with the samples and the RF of specific standards, allowed the identification of the following compounds:

EEBD: ferulic acid (C_10_H_10_O_4_), caffeic acid (C_9_H_8_O_4_), kaempferol (C_15_H_10_O_6_), rutin (C_27_H_30_O_16_) and quercetin (C_15_H_10_O_7_);

EEGP: ferulic acid, caffeic acid, rutin and quercetin.

Using this technique, it was not possible to assess the specific quantity of each compound, due to the similarity in chemical structure. However, the TLC method allowed to observe the presence of kaempferol in EEBD, which was not present in EEGP.

### Determination of total soluble solids

The total soluble solids present in EEGP were equal to 35 °Bx and the EEBD showed 25 °Bx. The value obtained for EEGP was equal to the minimum established by the Ministério da Agricultura e do Abastecimento (Brasil, 2001). This Brazilian public agency is responsible for regulating and certifying the quality of honey and propolis produced and sold in the country. By the results, the ethanolic extract of propolis produced and used in this study meets the quality requirements of national laws. Regarding the EEBD, there is no regulation because it is not used in folk medicine.

### Pre-treatment with ethanolic extracts

**Figure 3:**
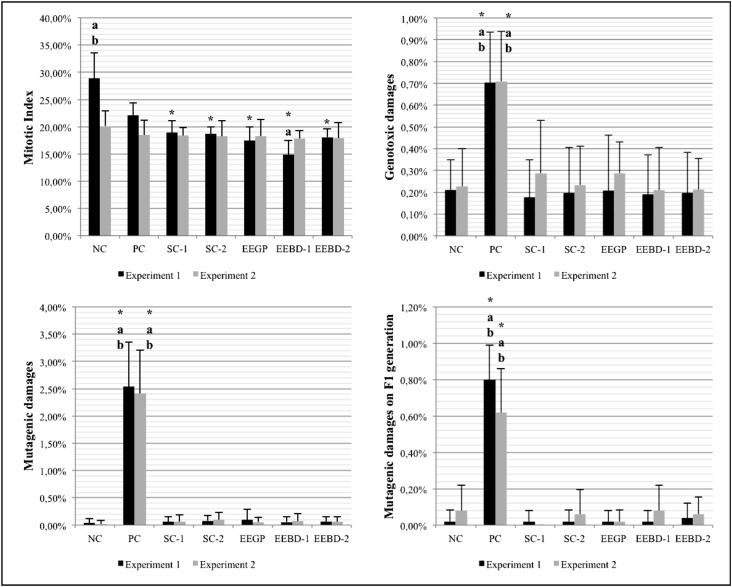
Results obtained from the first collection of *A. cepa* roots, which were used to evaluate the cytotoxic, genotoxic and mutagenic potential of the ethanolic extracts (pre-treatment). NC: negative control; PC: positive control; SC-1: solvent control 1 (ethanol – 0.042 μL/mL); SC-2: solvent control 2 (ethanol – 0.21 μL/mL); EEGP: ethanolic extract of Brazilian green propolis; EEBD-1: ethanolic extract of *Baccharis dracunculifolia* (0.06 μL/mL); EEBD-2: ethanolic extract of *Baccharis dracunculifolia* (0.30 μL/mL). *^,a,b^Statistically significant when compared to the NC, SC-1 and SC-2, respectively, by the Kruskal-Wallis/Dunn's test (p ≤ 0.05).

The data obtained from the first collection and the analysis of the respective slides is shown in Figure 3. Regarding the mitotic index (MI), considered a parameter of cytotoxicity, we conclude that this measurement was not consistent because of the variation presented between the two replicates for both extracts, and mainly for the negative control (NC). The data corroborate the information about MI as having, sometimes, a low efficiency as an endpoint of cytotoxicity, depending on the samples analyzed (Fernandes *et al.*, 2007). Thus, it was not possible to infer about the cytotoxicity of the ethanolic extracts in this study.

The results of genotoxic and mutagenic damages from the first collection showed, as expected, the efficacy of MMS as a positive control (PC) for the *Allium cepa* test system, when compared to the NC group. With respect to the solvent control (SC), none of the concentrations induced damages in meristematic or F1 generation cells. According to Phillips and Jenkinson (2001), several studies indicate that ethanol generally is not able to induce genotoxic/mutagenic effects, *in vivo* or *in vitro*. These effects are only positive if the ethanol concentration is too high, or when the assay is performed to assess chronic exposure.

Since the SC treatment did not induce any significant alterations when compared to the NC group, we suggest that the concentrations used in this work were safe to *A. cepa*. leading to the conclusion that the result obtained from the exposure to the ethanolic extracts only reflects the action of their own respective components.

The results of the first collection also showed that neither extract induced genotoxic or mutagenic effects. This data is in accordance with Nieva Moreno *et al.* (2005), who found the same result in their analysis when testing ethanolic extracts of Argentine propolis on *A. cepa*. In the *in vivo* tests performed by Tavares *et al.* (2006) the ethanolic extracts of propolis were genotoxic only in higher concentrations, like 100 μg/mL. Pereira *et al.* (2008) tested propolis in mice and also verified that DNA damages were induced only by concentrations higher than 1,000 mg/kg bodyweight.

Resende *et al.* (2007) evaluated the effects of extracts of *Baccharis dracunculifolia* on rat erythrocytes, and the authors observed no change in micronuclei frequency when compared to a NC, *i.e.*, the extracts did not induce mutagenicity.

### Clastogenic treatment

After the second collection, another alteration was observed between the mitotic indexes registered for the NC of both experiments, which excludes the possibility to assess the anticytotoxic potential of the EEGP and EEBD.

**Figure 4:**
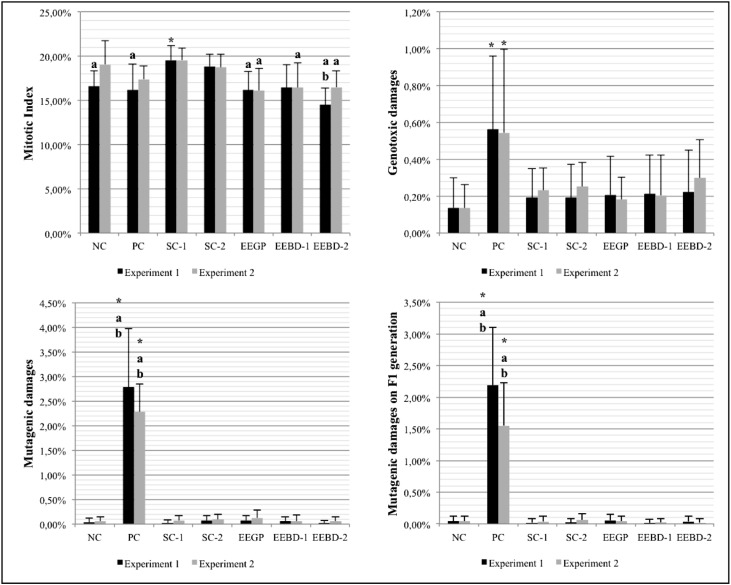
Results obtained by the second collection of *A. cepa* roots, which were used to evaluate the anticytotoxic, antigenotoxic and antimutagenic potential of the ethanolic extracts (clastogenic treatment with methyl methanesulfonate - MMS). NC: negative control; PC: positive control; SC-1: solvent control 1 (ethanol – 0.042 μL/mL); SC-2: solvent control 2 (ethanol – 0.21 μL/mL); EEGP: ethanolic extract of Brazilian green propolis; EEBD-1: ethanolic extract of *Baccharis dracunculifolia* (0.06 μL/mL); EEBD-2: ethanolic extract of *Baccharis dracunculifolia* (0.30 μL/mL). *^,a,b^Statistically significant when compared to the NC, SC-1 and SC-2, respectively, by the Kruskal-Wallis/Dunn's test (p < 0.05).

Furthermore, neither the EEGP nor the EEBD did jeopardize onion cells. Both were able to block DNA damages induced by MMS in this test system, as shown in Figure 4. Apparently, the two tested extracts protected the cells against the known clastogenic properties of this alkylating agent. In the assessment of the antigenotoxic potential, the frequency of chromosomal aberrations was statistically significant only for the PC group, when compared to the NC. The SC treatment did not harm meristematic or F1 generation cells, corroboration the hypothesis that ethanol did not altered cell homeostasis.

Both EEGP and EEBD inhibited MMS, used after the pre-treatment with the extracts, to induce genotoxic or mutagenic effects (Figure 4). When comparing the results from EEGP and EEBD, it is possible to see that EEGP and EEBD-1 were more effective in cell protection, since EEBD-2 showed a slight increase in the frequency of chromosomal aberrations.

Some researchers describe propolis as a compound that can reduce damages caused by mutagens. By testing several strains of *Salmonella typhimurium* (TA98, TA100 e TA102), Varanda *et al.* (1999) demonstrated, an antimutagenic activity exerted by ethanolic extract of propolis against daunomycin, benzo[a]pirene and aflatoxin. Jeng *et al.* (2000) performed *S. typhimurium* tests and inferred that the ethanolic extract of propolis was able to inhibit the mutagenic action of four mutagens (two with direct and two with indirect action). Another study, conducted by Fu *et al.* (2004), investigated the antimutagenic potential of propolis extract against daunomycin and aflatoxin by *S. typhimurium* tests and indicated the same effect for cyclophosphamide and mitomycin in mice.

Nieva Moreno *et al.* (2005) also reported that samples of propolis were capable of inhibiting the mutagenic effects of isoquinoline and of 4-nitro *o-*phenylenediamine, using *S. typhimurium* tests. Another study demonstrated the antigenotoxic and antimutagenic effects of ethanolic extract of propolis against the chemotherapy drug doxorubicin in Chinese hamster ovary (CHO) cells (Tavares *et al.*, 2006), and Russo *et al.* (2006) published a study in which they confirmed a protective effect after propolis application against DNA damages induced by benzo[a]pyrene and by reactive oxygen species in human spermatozoa. Thus, our results of *A. cepa* assays, based on the pre-treatment of onion roots with EEGP or EEBD followed by a clastogenic treatment, reinforce the view that propolis can act as an antimutagenic agent.

Although the effects of propolis are extensively studied, there is a lack of investigations testing the effects of extracts made with the main botanical source of propolis, in our case the plant *Baccharis dracunculifolia*. This plant and the respective type of propolis is endemic to a specific region of Brazil, located in the south of the state of Minas Gerais and in the north of the state of São Paulo (Alencar *et al.*, 2005). In this region, bees collect fragments of young leaves, buds and floral buds of "*alecrim-do-campo*" (*B. dracunculifolia*), a common plant belonging to the Brazilian savannah (cerrado) (Kumazawa *et al.*, 2003; Teixeira *et al.*, 2005).

Park *et al.* (2005) showed that, besides being the main source of raw material for bees in the synthesis of Brazilian green propolis, *B. dracunculifolia* can be used to make an ethanolic extract, which was responsible for inhibiting the toxicity of a dioxin, known for its powerful toxicity, with recognized carcinogenic and teratogenic potential. A study conducted by Resende *et al.* (2007) evidenced the antimutagenicity of the extract of *Baccharis dracunculifolia* against damages caused by doxorubicin, resulting in the reduction of chromosomal damages in mice. It is already known that extracts from this plant exhibit pharmacological properties that could be more explored, in addition or instead of propolis.

The frequencies of chromosomal aberrations (CA) were observed in meristematic cells from the roots pretreated with each extract and subsequently exposed to the MMS. This procedure was conducted to assess how these two compounds could possibly reduce the influence of the clastogenic agent. Since the extracts did not induce significant values of CA, it was possible to assess the percentages of damage reduction. Herein, EEGP was more effective, with 105.81% less damage when compared to the PC. And while the EEGP worked very well against genotoxicity, the EEBD concentrations were less active, but also functional: 101.78% for EEBD-1 and 87.90% for EEBD-2.

As detected for CA, the frequencies of micronuclei (MN) observed in meristematic and F1 cells from roots submitted to the same method of exposure were not statistically significant when compared to the NC. These results indicate that these compounds did protect the cells against the effects of MMS. By applying the formula of reduction of damages, similar efficiencies were noted for EEGP, EEBD-1 and EEBD-2. The reduction was 97.80% for EEGP, 99.76% for EEBD-1 and 102.01% for EEBD-2, based on the frequencies of the SC, PC and each extract. EEBD-2 was capable of reducing the frequency of MN to a level below that observed in SC and NC. Interpreting these results, it is possible to suggest that the ethanolic extract of propolis was an efficient antigenotoxic agent, while the ethanolic extract of *B. dracunculifolia* was more active as an antimutagenic agent, mainly in the higher concentration.

The mechanisms of action exhibited by the compounds of Brazilian green propolis and "*alecrim-do-campo*" extracts are not well understood. According to Tavares *et al.* (2006), the extract of propolis could have the characteristic of a "Janus" substance, *i.e.*, it could behave as a genotoxic and an antigenotoxic agent, depending on the experimental condition used. In their work, the extract was genotoxic when applied in high concentrations (100 μg/mL), but exerted chemopreventive activities when used in lower concentrations (12.5 μg/mL). In a study conducted by Mersch-Sundermann *et al.* (2004), this same effect was noted for the hydroethanolic extract of *Toxicodendron quercifolium*, which is widely used by homeopathy practitioners. The researchers revealed that flavonoids present in *T. quercifolium* extract, such as kaempferol and quercetin, can induce this same effect. Our analysis made by TLC identified kaempferol as a component in EEBD, while quercetin was identified as a component in both EEBD and EEGP. However, in the present work, neither the EEBD nor the EEGP showed genotoxic or mutagenic effects, maybe because they were used in concentrations lower than those recommended by the prescribing information of commercial extracts.

The biological effects of these extracts are derived from their phenolic compounds content, mainly due to the presence of flavonoids. Lima (Lima ROA, 2007, PhD Thesis, UNESP, Botucatu, Brazil) tested three substances found in Brazilian green propolis samples and detected an increase in MMS mutagenicity in a CHO cell culture. Nevertheless, these same substances induced a chemopreventive activity against two other mutagens.

Flavonoids are known as powerful antioxidants, with some of them also having prooxidant and mutagenic effects. Wattenberg (1985) was one of the first researchers to suggest that flavonoids could exert antimutagenic and anticarcinogenic effects. Furthermore, Tavares *et al.* (2006) suggest that flavonoids are the main components responsible for the mutagenic and antimutagenic effects of propolis.

Several classes of flavonoids contain a broad spectrum of biological activities, and it is believed that their specific cellular activity is related to their chemical structure (Middleton and Kandaswami, 1993). According to Horn and Vargas (2008), among several phytochemical compounds, flavonoids and tannins possess mutagenic and antimutagenic properties.

Agullo *et al.* (1996), in their work, tried to elucidate the importance of the structure-activity relationship of flavonoids and obtained interesting results. By comparing five families of flavonoids with chemical structure differences in a treatment of HT29 cells, the authors identified structural characteristics associated to cytotoxic activities. Apparently, flavonoids belonging to the flavones and flavonols classes are more potent and affect cellular viability, while those belonging to the catechins, flavanones and isoflavones classes are inactive. Casagrande and Darbon (2001) suggested that flavones, flavonols and isoflavones (except daidzein) are powerful antiproliferative substances, independent of the number of hydroxyl groups they have, since the C-ring of the flavonoid is preserved. For Agullo *et al.* (1996), the absence of unsaturation between C2 and C3 results in the loss of cytotoxicity. On the other hand, for Casagrande and Darbon (2001), the cytotoxicity could be weaker or even inexistent in the absence of this double bound (*e.g.* in flavanones and flavononols), and this lack of bond could be allied to the absence of the oxo function in position 4 of the C-ring, as is the case in catechins.

Choi *et al.* (1994) demonstrated that a methanolic extract of a flavonoid-rich plant exhibited antimutagenic effects, in contrast to other works that showed flavonoids as mutagens. Nevertheless, another study also showed that flavonoids could suppress and/or reduce the mutagenicity of several chemical compounds (Depeint *et al.*, 2002). Our results also showed that EEBD presents antimutagenic effects, which corroborates the data of the cited authors, who associated flavonoid-rich plant extract to an antimutagenic activity.

Steele *et al.* (1985) obtained negative results on the mutagenicity of catechins in assays performed with several bacterial strains. Under the same experimental conditions, these authors demonstrated that the same flavonoids also inhibited the mutagenic action of aromatic amines, which are known as carcinogenic substances. Furthermore, they described that the mechanism of action could involve a direct interaction between the mutagen and the catechin, and/or an indirect action in which the flavonoids could inhibit the formation of carcinogenic metabolites.

Despite many works about the antigenotoxic and/or antimutagenic potential of plant extracts, the underlying mechanisms of these effects are still unknown or not wholly explained. Antimutagenicity could be a result of antioxidant activity, or of the interference by one or more active compounds on metabolic routes, where mutagens could act (Resende *et al.*, 2007). Knasmüller *et al.* (2002) highlight that many chemopreventive compounds can act simultaneously, at different degrees of protection, making the explanation more difficult.

### Recovery assay

**Figure 5:**
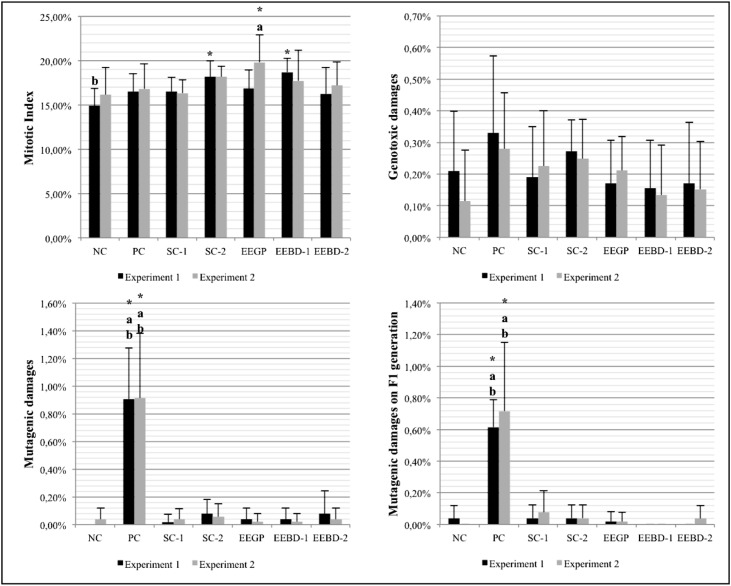
Results obtained with the third collection of *A. cepa* roots, which were used to evaluate the continuing effects of the ethanolic extracts over the cells (recovery assay). NC: negative control; PC: positive control; SC-1: solvent control 1 (ethanol – 0.042 μL/mL); SC-2: solvent control 2 (ethanol – 0.21 μL/mL); EEGP: ethanolic extract of Brazilian green propolis; EEBD-1: ethanolic extract of *Baccharis dracunculifolia* (0.06 μL/mL); EEBD-2: ethanolic extract of *Baccharis dracunculifolia* (0.30 μL/mL). *^,a,b^Statistically significant when compared to the NC, SC-1 and SC-2, respectively, by the Kruskal-Wallis/Dunn's test (p < 0.05).

By the recovery assay, it was possible to confirm that the mitotic index was not a good parameter for this study. Again, the results were different between both experiments, which makes this data unreliable (Figure 5).

Regarding genotoxicity, no treatment group showed a statistically significant difference when compared to the NC. Despite the increase found in some treatments after the recovery assay, no genotoxic damages were observed at high frequencies, even for the PC. This may indicate the *A. cepa* cells are capable of recovering from CA by simply removing them from the treatment and keeping them in a safe environment, such as ultrapure water. Therefore, it is possible to conclude that both, EEGP and EEBD, were still effective in protecting the onion cells, as these extracts showed the lowest frequencies of CA observed after the recovery assay. It also shows that MMS had a limited clastogenic effect, restricted to the exposure period, which means that if MMS stops to act on the cells, and the cells are still viable, they can recover from the damage and the organism can survive.

Concerning the results related to mutagenicity, only the PC showed significant differences when compared to the NC. As the PC group presented high frequencies of CA in the clastogenic treatment, we would expect this high frequency of MN, as DNA damages, like breaks or chromosomal losses, could result in MN (Fenech, 2000). As the extracts did prevent CA in clastogenic treatment, the occurrence of MN in meristematic cells and, mainly, in F1 cells, was not expected.

## Conclusion

In this work, the ethanolic extract of Brazilian green propolis and the ethanolic extract of *Braccharis dracunculifolia* did not induce genotoxicity or mutagenicity in the *Allium cepa* test system. In fact, they were effective in the protection of meristematic and F1 cells of *A. cepa* against the clastogenicity of methyl methanesulfonate. The plant bioindicator *A. cepa* was also a sensitive test system to detect the induced and the prevented damages of propolis extracts, and can be recommended to assess antigenotoxicity and antimutagenicity, as it generally has shown good correlation with Ames test, mammalian tests and human cell culture assays. The results here obtained reinforce the importance of natural products (*e.g.* propolis and its botanical source) as compounds that could be used in daily diet or in the formulation of pharmaceuticals and other personal care products, likely to enhance the protection of our body against the development of chronic diseases. Despite the higher flavonoid concentration in Brazilian green propolis, our results showed that the ethanolic extract of its source plant, *Baccharis dranunculifolia,* induced similar effects and it could be used as an alternative to substitute the Brazilian green propolis extract. Furthermore, the plant could be cultivated in greenhouses and could also be used to produce commercial extracts.

Since the number of published papers on the mechanisms of action of plant and propolis extracts are scarce, the need of more studies with these chemical compounds becomes evident, in order to clarify their mode of interaction with the cells.
